# Do Peers See More in a Paper Than Its Authors?

**DOI:** 10.1155/2012/750214

**Published:** 2012-11-27

**Authors:** Anna Divoli, Preslav Nakov, Marti A. Hearst

**Affiliations:** ^1^Pingar Research, Pingar, Auckland 1010, New Zealand; ^2^Qatar Computing Research Institute, Qatar Foundation, Tornado Tower, Floor 10, P.O. Box 5825, Doha, Qatar; ^3^School of Information, University of California at Berkeley, CA 94720, USA

## Abstract

Recent years have shown a gradual shift in the content of biomedical publications that is freely accessible, from titles and abstracts to full text. This has enabled new forms of automatic text analysis and has given rise to some interesting questions: How informative is the abstract compared to the full-text? What important information in the full-text is not present in the abstract? What should a good summary contain that is not already in the abstract? Do authors and peers see an article differently? We answer these questions by comparing the information content of the abstract to that in *citances*—sentences containing citations to that article. We contrast the important points of an article as judged by its authors versus as seen by peers. Focusing on the area of molecular interactions, we perform manual and automatic analysis, and we find that the set of all citances to a target article not only covers most information (entities, functions, experimental methods, and other biological concepts) found in its abstract, but also contains 20% more concepts. We further present a detailed summary of the differences across information types, and we examine the effects other citations and time have on the content of *citances*.

## 1. Introduction

Text mining research in biosciences is concerned with how to extract biologically interesting information from journal articles and other written documents. To date, much of biomedical text processing has been performed on titles, abstracts, and other metadata available for journal articles in PubMed ^1^, as opposed to using full text. While the advantages of full text compared to abstracts have been widely recognized [1–5], until relatively recently, full text was rarely available online, and intellectual property constraints remain even to the present day. These latter constraints are loosening as open access (OA) publications are gaining popularity and online full text is gradually becoming the norm. This trend started in October 2006, when the Wellcome Trust ^2^, a major UK funding body, changed the conditions of grants, requiring that “research papers partly or wholly funded by the Wellcome Trust must be made freely accessible via PubMed Central ^3^ (PMC) (or UK PubMed Central once established) as soon as possible, and in any event no later than six months after publication” [6]. Canadian Institutes of Health Research followed, as did the National Institute of Health (NIH) in the USA in April 2008. ^4^ Moreover, many publishers founded and promoted OA initiatives, namely, BioMed Central ^5^ (BMC) and the Public Library of Science ^6^ (PLoS). PubMed now offers access to all OA publications via PMC. The availability of OA publications has allowed several recent text mining and information retrieval competitions turning to use full-text corpora, for example, BioCreAtIvE since 2004, the TREC Genomics Track since 2006, and the BioNLP shared task since 2011.

The rise of full text, which differs in length (both overall length and average sentence length), structure (e.g., use of parenthesized text, tables, and figures), and content from abstracts, has posed many new challenges for biomedical text processing, for example, standard tools like part-of-speech and gene mention taggers were found to perform much worse on article bodies than on abstracts [7]. The availability of full text has further opened up some more general interrelated questions.

1. *How informative is the abstract compared to the full text?*
2. *What important information in the full text does not appear in the abstract?^7^*
3. *What should an ideal summary of the full text contain that is not already in the abstract?*
4. *What are the differences in the way authors and peers see an article?*

We explore these questions indirectly, using an underexplored information source: the sentences containing the citations to a target article or *citances*. While cocitation analysis is commonly-used for determining the popularity, and by association, the importance of a publication [8–15], our focus here is on the *contents* of the sentences containing the citations, that is, the citances.

In particular, we compare the information content of the abstract of a biomedical journal article to the information in all citances that cite that article, thus contrasting the important points about it as judged by its authors versus as seen by peer researchers over the years following its publication. Put another way, we use citances as an indirect way to access important information in the full text ^8^. The idea is that (1) any information not mentioned in the abstract but referred to in citances should be coming from the full text, and (2) entities and concepts mentioned in a citance should be important and somewhat representative of their source.

To give an example, here is the abstract of an article (PubMed ID 11346650):

*Multiple Mechanisms Regulate Subcellular Localization of Human CDC6.*

*CDC6 is a protein essential for DNA replication, the expression and abundance of which are cell cycle-regulated in Saccharomyces cerevisiae. We have demonstrated previously that the subcellular localization of the human CDC6 homolog, HsCDC6, is cell cycle-dependent: nuclear during G(1) phase and cytoplasmic during S phase. Here we demonstrate that endogenous HsCDC6 is phosphorylated during the G(1)/S transition. The N-terminal region contains putative cyclin-dependent kinase phosphorylation sites adjoining nuclear localization sequences (NLSs) and a cyclin-docking motif, whereas the C-terminal region contains a nuclear export signal (NES). In addition, we show that the observed regulated subcellular localization depends on phosphorylation status, NLS, and NES. When the four putative substrate sites (serines 45, 54, 74, and 106) for cyclin-dependent kinases are mutated to alanines, the resulting HsCDC6A4 protein is localized predominantly to the nucleus. This localization depends upon two functional NLSs, because expression of HsCDC6 containing mutations in the two putative NLSs results in predominantly cytoplasmic distribution. Furthermore, mutation of the four serines to phosphate-mimicking aspartates (HsCDC6D4) results in strictly cytoplasmic localization. This cytoplasmic localization depends upon the C-terminal NES. Together these results demonstrate that HsCDC6 is phosphorylated at the G(1)/S phase of the cell cycle and that the phosphorylation status determines the subcellular localization.*

And here are some citances pointing to it:

*Much of the soluble Cdc6 protein, however, is translocated from the nucleus to the cytoplasm when CDKs are activated in late G1 phase, thus preventing it from further interaction with replication origins [#C, #C and #TC].*

* To ensure that the pre-RC will not re-form in S or G2, Cdc6p is phosphorylated and degraded in yeast (#C; #C; #C) or exported to the cytoplasm in higher organisms (#TC; #C; #C; #C; #C).*

* It is phosphorylated by cyclin A-cdk2 at the G1-S transition and this modification causes some, but not all, of the Cdc6 to be exported out of the nucleus (#TC; #C; #C and #C). * **

* Cdc6CyΔ has a mutation in a cyclin binding motif that is an essential part of the substrate recognition signal for cdks (#TC).*

* After entry into S phase, phosphorylation of HsCdc6, probably by cyclinA/CDK2, leads to its export from nucleus to the cytoplasm via NES [#TC]. * **

* Once replication begins, Cdc6 is degraded in yeast (#C, #C, #C, #C, #C), whereas for mammals it has been suggested that Cdc6 is translocated out of the nucleus during S phase in a cyclin A-Cdk2- and phosphorylation-dependent manner (#C, #TC, #C,-#C, #C) and then subject to degradation by the anaphase-promoting complex (#C, #C, #C). * **

In the above examples, #TC refers to the publication we are comparing against (the target citation: PubMed ID 11346650), whereas #C refers to other publications. Throughout this paper, we will refer to these citation sentences to other publications as *adjoining citations*.

Previous studies have discussed some of the potential of the use of *citances* for literature mining [16, 17]. Similar to anchor text on the web (visible, clickable text in a webpage, clicking on which navigates the user to another webpage), they are votes of confidence about the importance of a research article. Collectively, they also summarize the most important points about the target article, which makes them a potential surrogate for its full text [18] and an important knowledge source when generating a survey of scientific paradigms [19].

While previous work has focused on the *words* in citances, we compare their contents to the contents of the abstracts using coarse-grained biologically meaningful *concepts* such as entities, functions, and experimental methods. Focusing on the area of molecular interactions, we perform careful *manual* analysis, and we present detailed summary of the differences across information types. We further study the effects that other citations and temporal measures have on the contents of citances. Finally, we verify these manual results with a large-scale automatic analysis.

In the remainder of this paper, we first discuss related work, then we describe our concept annotation scheme, we perform manual and automatic analysis, and we summarize the results, aggregating them over information types. Finally, we discuss the findings and we point to some promising directions for future work.

## 2. Related Work

In the bioscience literature, several studies focused on comparing the information structure of abstracts to that of full-text. Schuemie et al. [4], building on work by Shaw [3], looked into the density (the number of instances found divided by the number of words) of MeSH terms and gene names in different sections of full text articles. They found that the density was highest in the abstract and lowest in the Methods and the Discussion sections. They further found that nearly twice as many biomedical concepts and nearly four times as many gene names were mentioned in the full text compared to the abstract. In a related study, Yu et al. [2] compared abstracts and full text when retrieving synonyms of gene and protein names and found more synonyms in the former. A more comprehensive study on the structural and content difference of abstracts versus full text can be found in [7].

There has been extensive work on automatically generating an article abstract from full text, which studies the relationship between sentences in full text to those in abstracts [1, 5]. However, this work does not consider citances.

A lot of work on citation analysis has focused on citation links and counts, which have been used to determine the relative importance of publications within a field and to study the interaction between different fields [11–14, 20]. Today, this kind of analysis is at the core of a number of scholarly sites, including CiteSeerX ^9^, DBLP ^10^, Google Scholar ^11^, Microsoft Academic Search ^12^, ACM Digital Library ^13^, IEEE Xplore ^14^, ACL Anthology ^15^, and ArnetMiner ^16^, to mention just a few. There have been also specialized research tools for exploring citation networks, for example, [21].

In natural language processing (NLP), research has focused in a different and arguably more interesting direction, using citations as an (additional) information source to solve various text processing problems. The growing interest in the research community on the topic culminated in 2009 in a specialized workshop on Text and Citation Analysis for Scholarly Digital Libraries (collocated with the 2009 Conference on Empirical Methods on Natural Language Processing ^17^).

An early overview of this general research direction was presented by White [16], who described three main lines of research.

First, citation sentences can be *categorized*, for example, as conceptual versus operational, organic versus perfunctory, and so forth. For example, Teufel and Moens [22] identified and classified citations in scientific articles and used them as features for classifying noncitance sentences, for the purpose of text summarization.

Second, *context analysis* is concerned with identifying recurring terms in citances and using them to help solve information retrieval tasks. For example, Nanba et al. [23] used citances as features to help classify papers into topics. Similarly, Bradshaw [24] indexed articles with the terms in the citances that cite them. Mercer and Di Marco [25] applied a similar idea to biomedical documents. Tbahriti et al. [26] used paper cocitation as a similarity measure when evaluating a biomedical information retrieval system. Rosario and Hearst [27] demonstrated that using citances to a publication can yield higher accuracy compared to using other sentences for the problem of multiway relation classification, applied to the identification of the interactions between proteins in bioscience text. Similarly, Kolchinsky et al. [28] improved protein-protein interaction extraction using citation network features. Aljaber et al. [29] used citances text as an additional input to improve document clustering, and Aljaber et al. [30] used the text contained in citances as an additional information source to improve the assignment of Medical Subject Headings (MeSH) terms, which are commonly-used in PubMed and other databases administered by the National Library of Medicine.

The third line of research, according to White, is concerned with *citer motivation*, that is, with identifying the reason authors cite earlier work, and why some work is more cited than other. Lehnert et al. [31] created a taxonomy of 18 citation types, such as method, attribution, fact, example, critisism, and built a system to classify citations in these types. Similarly, Teufel et al. [32] annotated citation sentences from computational linguistics papers according to their rhetorical functions (e.g., contrast/comparison in goals or methods, contrast/comparison in results, weakness of cited approach, neutral description, etc.), and Teufel et al. [33] and Teufel and Kan [34] described algorithms to automatically assign such rhetorical functions.

Another informative early overview can be found in Nakov et al. [17], who also proposed the use of *citances* (they coined this neologism to refer to citation sentences) for bioscience papers for various semantic processing tasks, including summarization of target papers, synonym identification and disambiguation, and as a way to generate candidate sentences for manual curation. They further applied text paraphrase techniques to normalize the myriad forms of expression of citances in order to determine which of them express the same subsets of concepts. This last objective was later facilitated by the work of Schwartz et al. [35] using multiple sequence alignment and conditional random fields with posterior decoding.

More importantly, Nakov et al. [17] proposed to use citances as an information source for automatic summarization of the scientific contributions of a research publication, which is somewhat related to the idea of using the information in hyperlinks to summarize the contents of a web page [36, 37]. This direction has been explored by a number of researchers thereafter.

Schwartz and Hearst [38] hypothesized that in many cases, as time goes by, citances can indicate the most important contributions of a paper more accurately than its original abstract.

Qazvinian and Radev [39] used citation summaries and network analysis techniques to produce a summary of the important contributions of a research paper. A related technique for the same problem was proposed by Mei and Zhai [40], who relied on language modeling techniques. In a subsequent extension, Qazvinian and Radev [41] have proposed a general framework to pick the sentence(s) from a target paper that a citance in another paper is most likely referring to.

More closely related to the present work, Elkiss et al. [18] compared the information contained in the set of all citances citing a given biomedical paper and the abstract for that paper, using a lexical similarity metric called *cohesion*. They found significant overlaps but also many differences since citances focus on different aspects than abstracts.

Mohammad et al. [19] compared and contrasted the usefulness of abstracts and of citances in automatically generating a technical survey on a given topic from multiple research papers from the ACL Anthology. They found that while abstracts are undoubtedly useful, citances contain important additional information. They further noted that abstracts are author-biased and thus complementary to the broader perspective inherent in citances.

There has been also work that goes in the opposite direction: instead of trying to summarize a document using the textual content of multiple citances to it, Wan et al. [42] built a system that summarizes it using its full text in order to provide the reader with a summary relevant to a given citance in another document.

Hoang and Kan [20] introduced another interesting task: automatic related work summarization. Given multiple articles (e.g., conference/journal papers) as input, they created a topic-biased summary of related work that is specific to a given target paper.

Citations, citances, and links between them are similar to hyperlinks and hypertext on the web. Anchor text has been used in most search engines for indexing and retrieval of web pages. Applications of anchor text include identification of home pages of people and companies [43], classification of web pages [44, 45], Web crawlers [46], improved ranking of search results [47], and web page summarization [36]. See [24] for an overview of the uses of anchor text.

Our present work is more general and more quantitative than that in the above publications. First, we do not restrict ourselves to a particular application, while most work above was limited to, for example, summarization. Second, we study the degree of overlap between the information contained in abstracts and citances from a biomedical perspective focusing on molecular interactions and using biomedically meaningful semantic units (rather than words) such as entities, functions, dependencies, characteristics, locations, species, time, experimental methods, chemicals, and disorders. Third, we use and/or map our annotations to MeSH ^18^, a standardized hierarchical resource, thus allowing for further comparisons and applications. Fourth, we study the effect of time on the way papers are cited. We further investigate the effect of the presence of adjoining citations. Finally, we report the results from both small and focused manual analysis and from large-scale automatic analysis.

## 3. Methods

We performed small-scale detailed manual analysis and large-scale fully automatic comparison of the information contained in citances and abstracts.

In the manual analysis, we considered 6 abstracts from PubMed in the molecular interaction domain, published during 1996–2002, and 136 citances to them, which we carefully annotated with the mentions of entities, functions, experimental methods, and other biological concepts. More details about the dataset can be found in Table 1. We used this dataset to compare the set of concepts that appear in the abstract of an article to the set of concepts that appear in the citances to that article. We also looked at the concepts mentioned in the citances over a six-year period to study changes over time.

In the automatic comparison, we analyzed 104 journal publications in PubMed (this included the six articles used for the manual analysis), again from the molecular interaction domain, published during 1995–2002, which received a total of 11,199 citances in the period 1995–2005. We annotated the MeSH terms in the abstracts of these publications and in the corresponding citances, and we mapped these terms to broad biomedical concepts; then, we proceeded with the manual analysis. MeSH is a comprehensive controlled vocabulary created for the purpose of indexing journal articles and cataloging books in the life sciences, and it is commonly used for annotations in the biomedical domain. We chose MeSH for our automatic annotations because it is a formal established resource that has a relatively simple structure, allowing for intuitive, pragmatic analysis.

### 3.1. Data Selection

Our goal was to find articles that are highly cited and are in an area of biology that has attracted a lot of text mining interest. The “Molecular Interaction Maps” NIH website ^19^ lists a number of annotations and references for each interaction map that the site covers. We selected 104 target articles from the “Replication Interaction Maps” collection and used the ISI citation service ^20^ to find which articles cite the targets. We downloaded them and used the code developed by Nakov et al. [17] to extract the citances. We further collected the abstracts and the full text as well as the MeSH terms and the substances indexed by PubMed for these articles. Six of the 104 articles were used for manual analysis.

### 3.2. Manual Annotation

We performed detailed manual analysis of the mentions of various biologically meaningful concepts in the abstracts of the target six articles and in 136 citances to them. For one target article, we considered all 46 available (in our dataset) citances, and for another one, we selected a comparable number of 51 citances, whereas for each of the remaining four articles, we analyzed 10 randomly selected citances to ensure some variation. We annotated a total of 844 concepts in the six abstracts and 2,096 in the 136 citances. See Table 1 for more detail.

**Figure 1:**
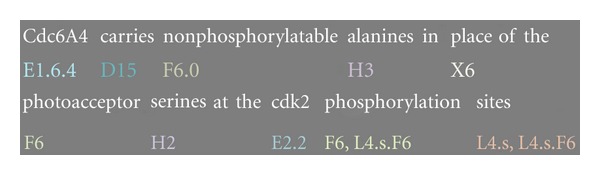
Example of an annotated citance. The citance is for PMID 11346650, demonstrating different categories of annotation (e.g., E, D; F; H…), subtypes (e.g., E1.64; L4.s; E2.2…), opposite concepts (e.g., F6.o), and complex IDs (e.g., L4.s.F6).

The goal of the annotation was to represent as much of the important contents of the citances as possible.  Table 2 describes the different types of concepts we annotate, and Figure 1 shows an example of an annotated citance.

Table 2 shows the categories for manual annotation. All datasets used in this study were annotated manually following a number of rules. Every unit (word or short phrase) was assigned an ID, and any matching unit within the same set was given the same ID. A few categories of units were decided for each set; they were reflected in the first part of the ID by a capital letter. The IDs, whenever possible, were very simple: composed of a single letter and a number. However, sometimes we tried to capture more complex units, for example, if “*Xenopus*" = “S1”, “*orc*” = "E1" and “*antibody*” = “E10”, then “*anti-Xorc1*” = “E10.E1.S1.1”, and if “*DNA*” = “H1” and “*synthesis*” = “F1”, then “*DNA synthesis*” = “H1.F1” so “*DNA*” is given the IDs: “H1, H1.P1” and “*synthesis*” is assigned “P1, H1.P1”. The last column shows the corresponding MeSH IDs, which were used for the automatic annotation.

We identified the distinct semantic units, words or phrases, and we assigned them annotation IDs, which had different prefixes (E, H, etc.) for different types of information. We assigned suffixes for subtypes (e.g., E2), and we represented complex concepts by combining IDs (e.g., E2.2). We used the same rules to annotate the citances (given below).

**Manual Annotation Rules**

1. Try to identify units (words or phrases) that convey information in one of the annotation categories (Table 2). Use words as annotation units, whenever possible.
2. Compare units by trying to match them to parts of other citances within the set.
3. If an entity (category E) is comprised of more than one word, consider the words as one unit and assign the same ID to each word.
4. Try to group entities together (extending to protein complexes and families) if used in the same context throughout the citances for a target document. Use subtypes when necessary to keep related concepts similarly labeled (.a,  .b, .c… or .1, .2, .3).
5. If an entity is complex, use "*·*" to join IDs, but keep the main entity in the front. For example, if *Xenopus* = S1, *orc* = E1 and antibody = E10, then the annotation for anti−*Xorc1* is E10.E1.S1.1 and for Xorc2 is E1.S1.2.
6. Annotate individual word units, but also consider complex concepts (e.g., *DNA replication*). Similarly to entities, capture concepts that are made of more than one unit by concatenating their IDs with “·”.
7. When annotating complex concepts, annotate each unit of the concept with the unit's ID followed by a comma, followed by the concept ID.
8. Consider *opposite* information units (e.g., competent-incompetent, increase-decrease). Capture these in the IDs by adding “.o”.
9. Consider subcategories of IDs by appending  .a, .b, … or .1, .2,… extensions if appropriate for the same citance set, for example, *prevent and inhibit*.

### 3.3. Data Analysis

The annotations of the citances and abstract sentences shown in Table 1 enabled us to run a number of comparisons between the content of the abstract and the corresponding citances, the outcomes of which are presented in the next section.

In our automatic analysis, we relied on MeSH, the U.S. National Library of Medicine's controlled hierarchical vocabulary. There are 15 main subtrees in MeSH, each corresponding to a major branch of the biomedical terminology, for example, subtree A corresponds to *anatomy*, subtree B to *organisms*, subtree C to *diseases*, and so forth. Down the MeSH hierarchy, concepts are assigned one or more positional codes, for example, A (*anatomy*), A01 (*body regions*), A01.456 (*head*), A01.456.505 (*face*), and A01.456.505.420 (*eye*). Note that MeSH is not a tree, but a lattice, and thus multiple paths are possible for the same concept, for example, *eye *is ambiguous, and it has one additional code: A09.371 (A09 represents *sense organs*).

We used an in-house MeSH term recognizer and normalizer tool, which we originally developed for our participation in the first Genomics Track [48], but which we significantly expanded thereafter. We used a version of the tool developed for the Second BioCreAtIvE Challenge [49]. The tool uses normalization rules in order to allow for the following variations in form: (1) removal of white space, for example, “*BCL 2*”⇒“*BCL2*,” (2) substitution of nonalpha-numerical characters with a space, for example, “*BCL-2*”⇒“*BCL2*,” and (3) concatenation of numbers to the preceding token, for example, “*BCL 2*”⇒“*BCL2*.” All possible normalizations and expansions of all known MeSH terms and their synonyms were generated offline and then matched against a normalized version of the input text using an exact, first-longest-string-matching measure. The matches were then mapped back to the original unnormalized text, and the corresponding MeSH IDs were assigned.

**Figure 2:**
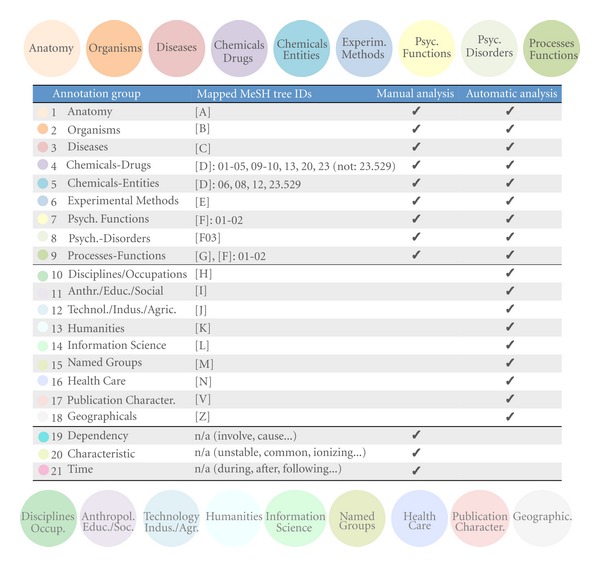
Semantic annotation groups. This figure depicts all different annotation types associated with abstract sentences and with citances. The overlap and, where possible, the mapping of automatic and manual annotations categories are also shown. See also Table 2 for details on the mapping of MeSH IDs to categories from the manual annotation.

Once the MeSH terms were identified, we considered (1) the whole MeSH tree ID and (2) the MeSH tree tag truncated to maximum 2 levels (xxx.xxx) in abstracts and citances^21^. We performed automatic analysis and mapping to identify different MeSH annotation groups (shown in Figure 2) and their counts in abstracts, corresponding citances, and their overlap. We also looked at annotations in citances with 0 adjoining citations (whose contents must have come from the target article) and how they compare to the annotations in abstracts. Finally, we looked at citances' annotations appearing in the same year as the original publication, as well as at additional/new annotations appearing in the following year, and additional annotations appearing 2, 3, and 4+ years later, and how they compare to annotations from the abstracts.

### 3.4. Category Mapping

There are a few distinct annotation categories in each manual and automatic schemata. However, for most categories of interest for the area of molecular interactions, the semantic annotations overlap. We provide the mapping in Figure 2.

## 4. Results

Here we describe the results of our manual and automatic analysis, trying to answer the research questions posed in the introduction. We further study the effect of the presence of adjoining citances and of the passage of time.

### 4.1. Differences between Abstracts and Citances

**Figure 3:**
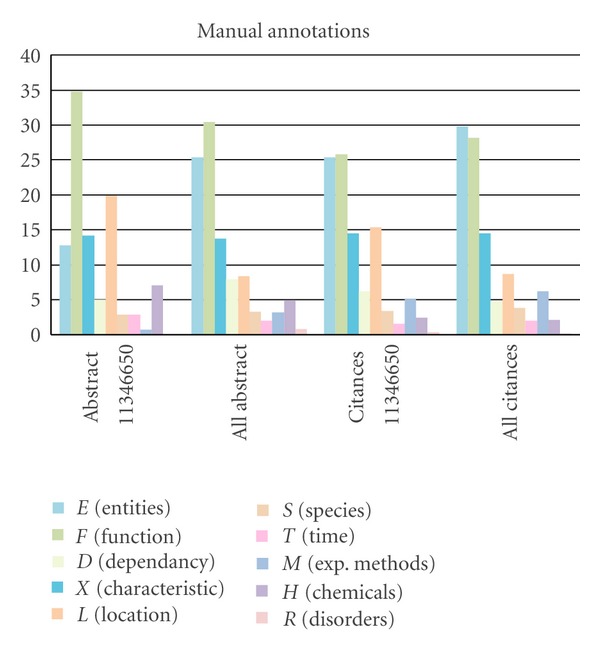
Distribution (in %) of the manually annotated categories for abstracts and citances. Shown are results for all abstracts and for the one with PubMed ID 11346650.

In order to examine the differences in the contents of abstracts and citances, we compared the distributions of the ten categories of concepts that we considered in the manual analysis (see Table 2).  Figure 3 shows these distributions (a) over abstracts and (b) over citances. It further presents these distributions (i) for all six articles, and (ii) for one article only, namely, the one with PubMed ID 11346650.

In Figure 3, we can see that there are generally higher proportions of “entities” and “experimental methods” annotations in citances than in abstracts. The difference for experimental methods was statistically significant for the two larger sets, corresponding to PubMed IDs 11346650 and 8939603.

**Figure 4:**
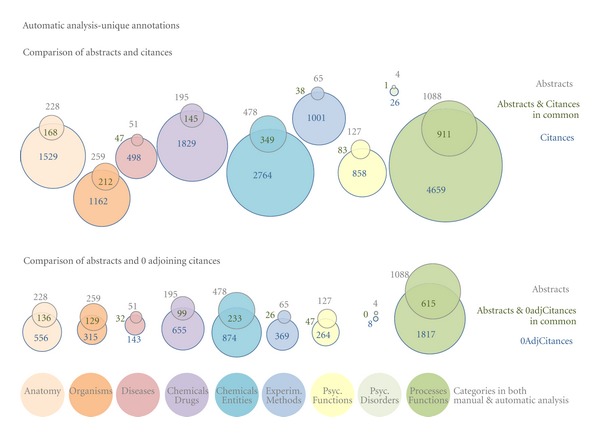
Number of unique concepts found in abstracts, all citances, and citances with 0 adjoining citations. Also shown is the overlap between all citances and abstracts.

**Figure 5:**
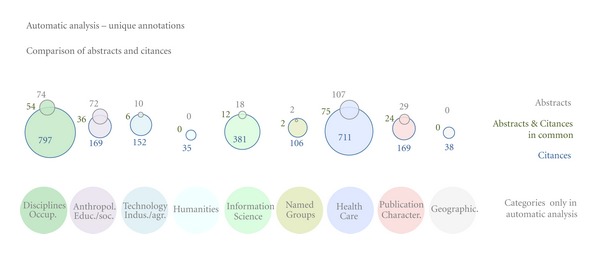
Unique Annotations found in abstracts, citances, and their overlap for the annotation categories defined only in the automatic analysis.

The top of Figures 4 and 5 use Venn diagrams to show the overlap of unique (i.e., each ID was counted just once regardless of how many times it actually occurred) semantic annotations between abstracts and citances for the large-scale automatic analysis.  Figure 4 shows the overlap over MeSH annotation categories that can be mapped (see Figure 2) to the manually assigned annotations, that is, those categories that were included in both the automatic and the manual analysis, whereas Figure 5 presents the overlap over annotation categories that were studied in the automatic but not in the manual analysis.

We see that indeed the categories in Figure 4, which we considered important for our dataset and used for the manual annotation, have a lot more unique annotations than the categories in Figure 5 that are largely less pertinent for molecular interactions (see Figure 2 for more details on the categories). We do see, however, that across all categories in both figures, citances carry a lot more annotations than abstracts with the overlap between the two being at least 50% of the abstract's unique annotations (with the exception of psychological disorders, representing a very small portion of the annotations). For most categories, the overlap is about 75–80%.

### 4.2. The Effect of Adjoining Citations and the Differences between Abstracts and Citances

Looking more closely at the data in Figure 3, we found that every annotation in our six manually annotated abstracts could be found in at least one citance. For the four articles for which we only consider 10 citances, we had to look for additional unannotated citances to get complete coverage for some of the concepts.

The contrary, however, was not true: some concepts found in citances were not mentioned in the abstract. Before describing this point in detail, we would like to note that very often in bioscience journal articles, a citation sentence backs up its claims with more than one reference. As we mentioned earlier, we call the references that appear in addition to the target *adjoining citations*. Our analysis has shown that citances containing adjoining citations are the source of most of that extra information. Thus, we decided to have a closer look at the clean cases of citances with zero adjoining citations (referred to as “zero adjoining citations” or “cit_0” below), that is, those that cited our target article only. Citances that refer to only one paper should really contain information that can be found in the citing paper.

In the manual analysis, we examined 23 citances with no adjoining citations, which corresponded to five of our target papers, and we found 73 distinct annotation types in the citances that did not appear in the abstracts. First, we checked whether the annotations conveyed biological meaning; if not, they were marked as “n/a.” Then we tried to find the extra annotations in the full text of the targets, and we examined the “MeSH/substances” that the target article was indexed with in PubMed. After all these checks, a few annotations were still “not found.” The distribution for each of the six articles is shown in Table 3.

**Figure 6:**
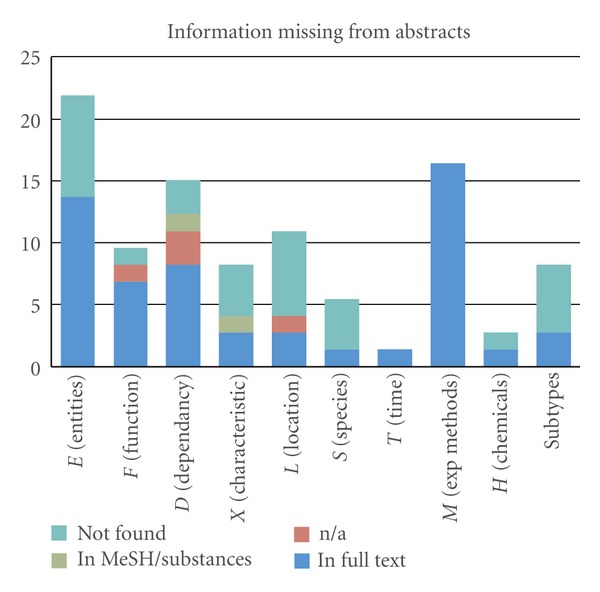
Categories of distinct manual annotation types not found in abstracts.

Table 3 and Figure 6 (manual evaluation) show that most of the concepts that abstracts do not contain fall under the entities or the experimental methods categories. Two others were mentioned in figures of the full text paper (PMID: 11298456) as part of describing an experimental technique. Two more were actually found in the full text (PMID: 8939603) as restriction enzymes, which are commonly used in experiments to cut *dsDNA*. Some other distinct annotation types missed by abstracts were also related to Methods, for example, *plasmid*, which was annotated as a chemical; in fact, plasmids are commonly-used in genetic engineering as vectors.

Some other entities had subtypes (e.g., *Wee1A*) and although the main type was matched in the full text, the specific subtype was not. In the species category, a sentence from cit_0 for the target PubMed ID 11251070 was referring to the animal category, which was not mentioned in the abstract. The full text mentioned *eukaryotes* and *various* organisms, but it was indexed with the more general MeSH term *animals*.

We further analyzed how adjoining citations affect the number of distinct annotation types by grouping the citances into five groups: cit_0, which cites the target paper only, cit_1/cit_2/cit_3, with one/two/three adjoining citations, and cit_4+, with four or more adjoining citations. In order to compare the effect of the adjoining citation, we took the abstract of each set (representing the minimum number of distinct annotation types), and we added each of the above groups separately as well as together (the abstract and the citances representing the maximum number of distinct annotation types). The results are shown in Table 4. We can see that the more references a citance has, the more distinct the annotation types that are introduced. The effect is most clearly pronounced for the two papers with a larger set of citances, those with PubMed IDs 8939603 and 1346650.

We also studied the effect of the adjoining citations in the larger dataset, which we used for the automatic analysis.  Figure 4 shows the effect that adjoining citations have on the semantic annotation content of citances. We can see that “zero adjoining citances” contain much less annotations in comparison to all citances, but the overlap of annotations with the abstracts' annotations are, proportionately, much larger.

### 4.3. The Effect of Time

Next, we studied how the concepts mentioned in the citances changed over time. For each target article in our large dataset, we grouped the citances per year of citation, from cited in the same year of publication to cited up to 4+ years thereafter.

**Figure 7:**
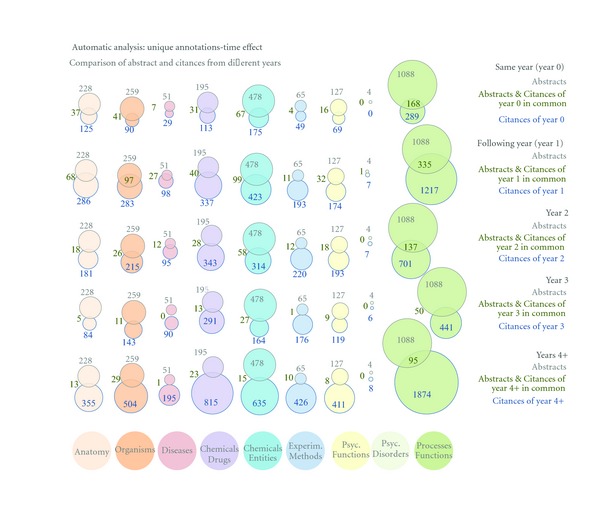
The effect of time. We show the unique semantic categories mentioned in the citances from the same publication year as the original target paper and how they overlap with the semantic categories matched in the target abstracts. Semantic annotations and overlap with the abstract for the following 1, 2, 3, and 4+ years are also shown. Note that only *new* unique semantic annotations are counted, for example, annotations of “citances of year 2” do not include any annotations that already appeared in years 0 or 1.

Our results (see Figure 7) show that with every year passing, new annotations are being assigned to the target paper via its citances. The majority of citances' annotations that overlap with abstracts' annotations appear within the first couple of years, but more are constantly added each following year. This is quite uniform across all categories. It would be of interest to conduct more in-depth analysis to see if these new annotations are representative of the research trends progression across the biomedical literature.

## 5. Discussion

In this section, we discuss the effect of the internal structure of the sentences on our methodology. We further provide a critical overview of our combination of manual and automatic analysis. Finally, we discuss the significance of our results and how they can be applied in a number of areas aiming at improving literature-mining solutions for life sciences research.

### 5.1. The Internal Structure of Citances

As we have seen above, the relationship between citances and citations is not always 1 : 1, for example, in some cases, a citance would contain citations to multiple target articles. While we acknowledged and analyzed the issue, we still treated citances as *atomic* from the viewpoint of the target article(s), assuming that the whole citance was commenting on it/them. Things are more complicated though: it is often the case that only part of a citance is really relevant. This is similar to HTML pages, where only part of a sentence containing a hyperlink is actually included in the hyperlink. Unfortunately, research publications, unless published in some hyperlink-friendly format, do not use such precise mechanisms for pointing out the relevant part of a citance. Yet, authors of research articles do use citations that refer to part of a citance, which poses interesting challenges to research on citances. See [50] for an overview. Below we list and illustrate three ways in which authors use references:

**Type 1**

Use separate citations for different parts of the citance.

**Example 5.1**

Subsequently, it has been observed that a similar motif is present also in substrates like Cdc6 [21] and retinoblastoma family of proteins [22] and the activator Cdc25A [13].

**Type 2**

Use citation(s) for part of the citance only.

**Example 5.1**

The nucleosolic or nonutilized Cdc6 then could either be translocated to the cytoplasm (10, 11, 16, 28, 33) or have its affinity for chromatin reduced but still remain in the nucleus (as our immunohistochemical and biochemical data would suggest); this would prevent inappropriate pre-RC formation and reinitiation of DNA replication.

**Type 3**

List multiple references together at the end of the citance.

**Example 5.1**

These and other biochemical and genetic studies in Drosophila and Xenopus demonstrate that the ORC functions in chromosomal DNA replication in multicellular eukaryotes, just as it does in yeast (25, 28–30, 48, 49).

Citances of Type 2 might have been the reason that a number of biological concepts mentioned in citances were not found in the full text of the target citations. Additionally, we could have used citances of Type 1 to detect more accurately the origin of the information in citances.

Notwithstanding that having considered this variation in citance structure would had enabled us to determine the source of information more accurately, as we discussed in the related work section, a lot of work has been done on the basis that references that appear together are related. Therefore, any additional information from other references can be used to augment the information from the target citation.

Finally, we should note that even knowing when a sentence contains a citation is a challenging task by itself since citation markers can differ in style. Moreover, even after a citation has been identified in text, resolving its target article is not a trivial task. For a further discussion on these issues, see [51–53].

### 5.2. Combining Manual and Automatic Analysis

We strived to map the categories of our manual schema to the automatic annotation schema the best possible way, while keeping them pertinent to the area of molecular interactions. Despite the significant overlap between these two schemata, the mapping was not ideal, as Figure 2 shows. For example, we could not use MeSH to automatically generate concepts covering events and relations, which were present in the manual annotation. To compensate for this, we added a number of additional concept categories that were easy to identify in MeSH, for example, disciplines, humanities, healthcare, and so forth (see Table 2, Figure 2).

Another issue with the automatic analysis was that the 1 : 1 mapping to the concept categories for the manual annotation was not possible since MeSH categories did not always align perfectly to our concepts. On the positive side, we relied on MeSH, which is a standard resource that is widely used in biomedical text mining. It provides many variants and synonyms for the concepts it covers, which allows us to handle the variety in expression that is inherent in natural language. Moreover, the MeSH concepts are organized in a hierarchical structure, which allows for a very easy mapping of whole subtrees to predefined categories; in the ideal case, all that is needed to define the mapping is to find the correct level of generalization in MeSH.  Table 2 shows how this was done in our case.

### 5.3. Using Semantic Annotations Found in Citances to Augment Annotations in Abstracts

While studying the effect of adjoining citations, we found that the majority of citances' unique annotation IDs that overlap with unique annotations found in the original target abstract can indeed be found in citances with 0 adjoining citations. This means that citances that cite multiple papers can be used to complement the abstract of each citing paper with more annotations. Imagine that an abstract with 20 semantic annotations assigned to it has 0adj_citances with 30 annotations and 15 of them overlap with the abstract annotations. Now, we have 15 more annotations that can be mapped to the abstract. The target paper is about to have 1+adj_citances that can be associated with a larger number of annotations, say 60; these new annotations can now also be associated with the original paper.

Much like modern media's social boosting from users assigning tags, these new annotations provided by expert peers can be used to help various NLP tasks. Here we propose utilization of these annotations for document summarization, document ranking, and automatic biological database annotation.

In the case of document summarization where most related work has concentrated on, we observe the following opportunities (1) A way to expand information by combining (union) the citances, which contain the best representative information from the full text (rich peer-produced resource), with the abstract (author-produced resource)—this would offer the best complete, inclusive summary. (2) A way to narrow down the information by using the intersection of the information found in citances and abstracts, especially years later—this would offer the most distilled, concentrated summary. (3) A way to generate a summary for a paper, even when its abstract and/or full text are not available in electronic form—that is, use just the citances.

In the case of document/sentence ranking, the density of these annotations in a sentence (or, alternatively, the category/type of annotation, or the relationship of the annotations to the original source) can be used to boost a weight-based ranking system.

Furthermore, our approach can be extended to other standardized resources (e.g., GO and UMLS) that are often used in biomedical databases to automatically map normalized entities and concepts to each other as well as to articles.

### 5.4. The Four Questions

Let us now go back to the four original research questions, keeping in mind that our dataset focused on molecular interactions, a very hot area for literature mining, as it is the main resource for constructing molecular networks and thus answering systems biology questions.

1. *How informative is the abstract compared to the full text?* We have shown that the information contained in the abstract and in the citances overlap to a large extent. Yet, there is information in the full text that is important enough to be referred to in citances, but it is not included in the abstract. Thus, abstracts cannot substitute the full text since peers cite information from the full text that is not always included in the abstract.
2. *What important information in the full text does not appear in the abstract?* We have shown that citances contain additional information that does not appear in abstracts. Since this information appears in a citance, then (1) it should come from the full text, and (2) it should be seen by peers as important. We studied several categories of biologically meaningful concepts and we found that citances contained more information for each of these categories; still, the differences were most pronounced for biological entities and experimental methods.
3. *What should a summary of the full text contain that is not already in the abstract?* —We believe that a good summary of an article should combine the information from its abstract and from citances. Citances give the viewpoint of multiple peers and are thus a very valuable information source. Our study has found that citances tend to mention more biological entities and to care more about experimental methods than authors do in their abstracts. Thus, we would recommend that summaries pay more attention to molecular entities and even consider including information on methods.
4. *What are the differences in the way authors and peers see an article?* Authors' viewpoint is summarized in the articles abstract, while peers' viewpoint is reflected in the citances to that article. Thus, articles are author-biased, while the set of citances, which are produced by many peers, is more objective. Moreover, citances are written years after the article was published, which also contributes to a more objective view to the contribution of an article: we have seen that, in the first year peers largely agreed with the authors, while differentiation was observed later when the citances have become arguably more divergent in content than the original target paper. The overlapping information though (found both in abstracts and in citances from years later) can be perceived as the most interesting, as it remains pertinent scientifically years later. Overall, we have found that authors focused in their abstracts on a smaller number of concepts compared to their peers. Moreover, peers tended to pay more attention to experimental methods compared to authors.

### 5.5. Future Directions

In future work, we would like to do a more careful study that would cover more and finer-grained categories in MeSH; trying resources like UMLS and GO is another attractive option. Looking at facts of larger granularity than just concepts, for example, looking at predicate-argument relations is another interesting direction for future work. We further plan to analyze the internal structure of citances, so that we can identify which part of the citance is relevant to a given citation. It would be also interesting to try similar analysis for other disciplines and areas of science, where the way research publications are written and the number of citations a publication receives may differ a lot from what we observe in life sciences.

Another interesting aspect is the passage of time. We have seen that while early citations tend to agree with the authors, later ones tended to diverge more from the original abstract. It would be interesting to see whether this means that later citations are really more objective. An important tool in this respect would be to look at the repetitiveness of citations, which we ignored in our present study, where we focused on unique concept mentions instead: if many peers stated the same fact, then maybe it should be deemed not only more important, but also more objective. Peer motivation for citing an article is important as well, for example, citations that cite a fact would probably agree with the abstract more than those that criticize it.

Last but not least, we are interested in using citances to help NLP applications. While previous work has already shown a number of such examples including information retrieval [24, 25], document summarization [19, 39, 40], document categorization [23], document clustering [29], MeSH terms assignment [30], relation extraction [27], and automatic paraphrasing [17], we believe that this list can be extended significantly.

## 6. Conclusion

Citances tell us what peers see as contributions of a given target article, while abstracts reflect the authors viewpoint on what is important about their work. Unlike citances, which typically focus on a small number of important aspects, abstracts serve a more general purpose: they not only state the contributions, but also provide a summary of the main points of the paper; thus, abstracts tend to be generally broader than citances. Yet, our manual and automatic comparison of abstracts and citances for articles describing molecular interactions has shown that, collectively, citances contain more information than abstracts.

We performed manual evaluation, which revealed that while all concepts in an article's abstract could be found in the citances for that article (provided that the article has already accumulated enough citations), the reverse was not true: citances mentioned about 20% more concepts than abstracts. Assuming that any information that is not mentioned in the abstract but is important enough to be referred to in citances should be coming from the full text, we can conclude that full text contains important information that is not mentioned in the abstract. We did not detect any significant changes in concept mentions over time.

The automatic analysis verified the results of the manual analysis on a larger scale, using MeSH terms, which were automatically mapped to the biological concepts from the manual analysis. These experiments confirmed our findings that most concepts mentioned in abstracts can be also found in citances. They further confirmed that citances contained some additional information, which in our case was primarily related to biological entities and experimental methods. The large-scale analysis has shown that the manual analysis could indeed be automated; the approach can be extended to other commonly-used biomedical resources such as GO and UMLS, which allow for uniform representation of concepts, that is, useful information about the semantic relationship between abstracts and citation sentences and among concepts themselves.

Overall, our results show that citances are good surrogates of the information contained in a biomedical journal article. The set of all citances citing a given research publication can be seen as concise summaries of its important contributions and thus using them can be preferable to the full text in a variety of scenarios. For example, they allow text mining applications to concentrate on potentially useful sentences without the need to deal with the full text, which is long, has a complex structure, and often would not be available at all, for example, for older publications. Since our work was based on biologically meaningful semantic concepts, it provides quantitative justification of their usefulness for text mining as it has been observed in previous work [17, 27, 30].

We can conclude that, with the recent growth of free access to journal articles and open access publications, full text should be seriously considered for yet another reason: it contains citances with information on the publications referenced therein. Peers cite (mention and comment) information that they see as important even if it is not mentioned in the original publication's abstract. We would further like to draw special attention to citances, as a good source of concise, verifiable information on molecular interaction networks. To answer the question posed by our title “Do Peers See More in a Paper than its Authors?”: yes they do, and we should leverage this information.

## Supplementary Material

Supplement Material 1: Excel file with the manual annotation data.
Supplement Material 2: Log file listing all the data used for the automatic annotation.
Supplement Material 3: Annotation counts for all categories used in the automatic analysis.

## Figures and Tables

**Table 1:** Summary of the data used for the manual analysis.

PubMed ID Of the target	Year of publication	Number of sentences analyzed		Number of annotations in		Number of papers the citances are derived from
		Abstract	Citances	Abstract	Citances
8939603	1996	17	51	192	728	27
11346650	2001	11	45	141	761	24
11125146	2000	8	10	91	144	10
11251070	2001	12	10	142	128	10
11298456	2001	9	10	146	178	9
11850621	2002	8	10	132	157	8

All		65	136	844	2096	88

**Table 2:** Categories used in the manual annotation.

Categories	Description	Examples	MeSH Tree IDs
E (entities)	Genes and proteins	MCM, protein, ORC, Skp2	D06, D08, D12, and D23.529
F (function)	Biological function or process	Regulation, pathway, and function	G, F01, F02
D (dependency)	Relationship type	Involve, cause	N/A
X (characteristic)	Modifier	Unstable, common, and ionizing	N/A
L (location)	Cellular or molecular part	C-terminal, cytosol, and motif	A
S (species)	Any taxonomic description	Human, mammal, and *S. cerevisiae *	B
T (time)	Temporal information	During, after, and following	N/A
M (exp methods)	Methods and their components	Recombination, transfect	E
H (chemicals)	Not including genes/proteins	DNA, thymidine, and phosphoryl	D (except: D06, D08, D12, and D23.529)
R (disorders)	Names and associated terms	Cancer, tumor, and patient	C, F03
Special Types:			
IDs with subtypes	Subtype of a BASIC type	Retain-change, common-distinct	
IDs with opposite	Opposite of a BASIC type	Cell cycle—G phase, CDK–CDK2	
Complex IDs	Combination of BASIC types	Radio-resistant DNA synthesis	

**Table 3:** Comparison of the number of distinct annotation types in abstracts and citances with zero adjoining citations. We used all sentences from the 6 abstracts and all 23 citances that were only citing one paper for this analysis.

PubMed ID	Abstract	Abstract and citances_0	Difference	n/a	In full text	In MeSH or substances	Not found
8939603	52	65	13	1	10		2
11346650	52	75	23	3	14		6
11251070	57	73	16	2	3	2	9
11298456	60	71	11		6		5
11850621	61	71	10		9		1

Total	282	355	73	6	42	2	25

**Table 4:** Number of citances with a different number of adjoining citations in each article and the number of distinct annotation types they contain. These statistics are for the manual analysis. For the automatic analysis, see Figure 4 and the supplementary material.

PMID	Citance number						Distinct annotation types (abstract and citances)					
	All cit.	Cit_0	Cit_1	Cit_2	Cit_3	Cit_4+	All cit.	Cit_0	Cit_1	Cit_2	Cit_3	Cit_4+
8939603	51	3	8	12	10	18	121	65	68	63	87	85
11346650	45	7	3	4	7	24	170	75	66	66	73	144
11125146	10	0	6	3	1	0	80		67	65	43	
11251070	10	7	0	0	0	3	88	73				73
11298456	10	3	3	2	0	2	96	71	72	66		70
11850621	10	3	4	1	0	2	98	71	76	67		71

Total	136	23	24	22	18	49	653	355	349	327	203	443

## Endnotes

1. http://www.ncbi.nlm.nih.gov/pubmed/
2. http://www.wellcome.ac.uk/
3. http://www.ncbi.nlm.nih.gov/pmc/
4. http://grants.nih.gov/grants/guide/notice-files/NOT-OD-08-033.html
5. http://www.biomedcentral.com/
6. http://www.plos.org/
7. Our study also helps answer the question: *what abstract claims are not (strongly) supported by the full text*? We hypothesize that these would be those claims that are cited very infrequently or not cited at all, but a separate study is required to answer this question.
8. Note that here we assume that peers base their citations on full text and not only on the abstract. While this is a strong assumption, we believe that it generally holds in the research community. Our previous studies have shown that biomedical researchers like to verify reported results, for example, by looking at the methods that were used and by exploring the images and the tables in the full text. This has also motivated us to create a specialized search engine, the BioText Search Engine (http://biosearch.berkeley.edu/), for searching the figures and tables contained in open access journals, which is described in [54, 55].
9. CiteSeerX: http://citeseer.ist.psu.edu
10. DBLP: http://www.informatik.uni-trier.de/~ley/db/
11. Google Scholar: http://scholar.google.com/
12. Microsoft Academic Search: http://academic.research.microsoft.com/
13. ACM Digital Library: http://dl.acm.org/
14. IEEE Xplore Digital Library: http://ieeexplore.ieee.org/Xplore/
15. ACL Anthology: http://aclweb.org/anthology-new/
16. ArnetMiner: http://arnetminer.org/
17. EMNLP 2009: http://conferences.inf.ed.ac.uk/emnlp09/
18. http://www.nlm.nih.gov/mesh/
19. http://discover.nci.nih.gov/mim/index.jsp
20. http://isiknowledge.com
21. The data on the analysis considering the extended tree IDs can be found in the supplementary material available online at doi:10.1155/2012/750214. The majority of results discussed in this paper refer to higher MeSH level annotation representing broader entities and concepts.
