# Supplementary material for: Do Peers See More in a Paper Than Its Authors?
Source: Adv Bioinformatics. 2012 Nov 27;2012:750214. doi: 10.1155/2012/750214 (PMC3514807; doi:10.1155/2012/750214)
Supplement: Supplementary file 3 [file 750214.f3.pdf]

**Categories**	**Description**	**Examples**	**MeSH Tree IDs**
E (entities)	genes & proteins	MCM, protein, ORC, Skp2	D06, D08, D12, D23.529
F (function)	biological function or process	Regulation, pathway, function	G, F01, F02
D (dependency)	relationship type	involve, cause	N/A
X (characteristic)	modifier	unstable, common, ionizing	N/A
L (location)	cellular or molecular part	C-terminal, cytosol, motif	A
S (species)	any taxonomic description	human, mammal, S. cerevisiae	B
T (time)	temporal information	during, after, following	N/A
M (exp methods)	methods & their components	recombination, tranfect	E
H (chemicals)	not including genes/proteins	DNA, thymidine, phosphoryl	D (except: D06, D08, D12, D23.529)
R (disorders)	names & associated terms	cancer, tumor, patient	C, F03

**MeSH Tree Structures - 2009**	**Closer Manual Category**
1. Anatomy [A]	L (location)
2. Organisms [B]	S (species)
3. Diseases [C]	R (disorders)
4. Chemicals and Drugs [D]	* H (chemicals) & E (entities)
5. Analytical, Diagnostic and Therapeutic Techniques and Equipment [E]	M (exp methods)
6. Psychiatry and Psychology [F]	** F (function) & R (disorders)
7. Phenomena and Processes [G]	F (function)
8. Disciplines and Occupations [H]	N/A
9. Anthropology, Education, Sociology and Social Phenomena [I]	N/A
10. Technology, Industry, Agriculture [J]	N/A
11. Humanities [K]	N/A
12. Information Science [L]	N/A
13. Named Groups [M]	N/A
14. Health Care [N]	N/A
15. Publication Characteristics [V]	N/A
16. Geographicals [Z]	N/A
* Inorganic Chemicals [D01] +	H (chemicals)

	Organic Chemicals [D02] +	H (chemicals)
	Heterocyclic Compounds [D03] +	H (chemicals)
	Polycyclic Compounds [D04] +	H (chemicals)
	Macromolecular Substances [D05] +	H (chemicals)
	Hormones, Hormone Substitutes, and Hormone Antagonists [D06] +	E (entities)
	Enzymes and Coenzymes [D08] +	E (entities)
	Carbohydrates [D09] +	H (chemicals)
	Lipids [D10] +	H (chemicals)
	Amino Acids, Peptides, and Proteins [D12] +	E (entities)
	Nucleic Acids, Nucleotides, and Nucleosides [D13] +	H (chemicals)
	Complex Mixtures [D20] +	H (chemicals)
	Biological Factors [D23] +	
	Antigens [D23.050] +	H (chemicals)
	Biological Markers [D23.101] +	H (chemicals)
	Blood Coagulation Factor Inhibitors [D23.113] +	H (chemicals)
	Blood Coagulation Factors [D23.119] +	H (chemicals)
	Chemotactic Factors [D23.125] +	H (chemicals)
	Inflammation Mediators [D23.469] +	H (chemicals)
	Intercellular Signaling Peptides and Proteins [D23.529] +	E (entities)
	Pheromones [D23.641] +	H (chemicals)
	Pigments, Biological [D23.767] +	H (chemicals)
	Toxins, Biological [D23.946] +	H (chemicals)
	Biomedical and Dental Materials [D25] +	H (chemicals)
	Pharmaceutical Preparations [D26] +	H (chemicals)
	Chemical Actions and Uses [D27] +	H (chemicals)
**	Behavior and Behavior Mechanisms [F01] +	F (function)
	Psychological Phenomena and Processes [F02] +	F (function)
	Mental Disorders [F03] +	R (disorders)
	Behavioral Disciplines and Activities [F04] +	N/A

2. Counts Summary

Looking at all abstracts, the following unique IDs where found (first row -> number, second row -> %):
Total number of unique annotations = 2829

A	B	C	D	Dchem	Dent	E	F	Ffunc	Fdis	G	H	I	J	K	L	M	N	V	Z
228	259	51	673	195	478	65	153	127	4	1088	74	72	10	0	18	2	107	29	0
8.06	9.16	1.80	23.79	6.89	16.90	5.41	5.41	4.49	0.14	38.46	2.62	2.55	0.35	0.00	0.64	0.07	3.78	1.03	0.00

Looking at all citances, the following unique IDs where found (first row -> number, second row -> %):
Total number of unique annotations = 17233

A	B	C	D	Dchem	Dent	E	F	Ffunc	Fdis	G	H	I	J	K	L	M	N	V	Z
1529	1162	498	4593	1829	2764	1001	962	858	26	4659	797	440	152	35	381	106	711	169	38
8.87	6.74	2.89	26.65	10.61	16.04	5.58	5.58	4.98	0.15	27.04	4.62	2.55	0.88	0.20	2.21	0.62	4.13	0.98	0.22

Abstracts and citances have the following unique IDs in common (first row -> number, second row -> %):
Total number of unique annotations = 2197

A	B	C	D	Dchem	Dent	E	F	Ffunc	Fdis	G	H	I	J	K	L	M	N	V	Z
168	212	47	494	145	349	38	101	83	1	911	54	53	6	0	12	2	75	24	0
7.65	9.65	2.14	22.49	6.60	15.89	4.60	4.60	3.78	0.05	41.47	2.46	2.41	0.27	0.00	0.55	0.09	3.41	1.09	0.00

Unique IDs found in abstracts but not in citances (first row -> number, second row -> %):
Total number of unique annotations = 632

A	B	C	D	Dchem	Dent	E	F	Ffunc	Fdis	G	H	I	J	K	L	M	N	V	Z
60	47	4	179	50	129	27	52	44	3	177	20	19	4	0	6	0	32	5	0
9.49	7.44	0.63	28.32	7.91	20.41	8.23	8.23	6.96	0.47	28.01	3.16	3.01	0.63	0.00	0.95	0.00	5.06	0.79	0.00

Unique IDs found in citances but not in abstracts (first row -> number, second row -> %):
Total number of unique annotations = 15036

A	B	C	D	Dchem	Dent	E	F	Ffunc	Fdis	G	H	I	J	K	L	M	N	V	Z
1361	950	451	4099	1684	2415	963	861	775	25	3748	743	387	146	35	369	104	636	145	38
9.05	6.32	3.00	27.26	11.20	16.06	5.73	5.73	5.15	0.17	24.93	4.94	2.57	0.97	0.23	2.45	0.69	4.23	0.96	0.25

Looking at all abstracts, the following truncated to max 2 levels unique IDs where found (first row -> number, second row -> %):
Total number of unique annotations = 1924

A	B	C	D	Dchem	Dent	E	F	Ffunc	Fdis	G	H	I	J	K	L	M	N	V	Z
193	200	51	351	156	195	57	150	124	4	630	68	66	10	0	16	2	101	29	0
10.03	10.40	2.65	18.24	8.11	10.14	7.80	7.80	6.44	0.21	32.74	3.53	3.43	0.52	0.00	0.83	0.10	5.25	1.51	0.00

Looking at all citances, the following truncated to max 2 levels unique IDs where found (first row -> number, second row -> %):
Total number of unique annotations = 7862

A	B	C	D	Dchem	Dent	E	F	Ffunc	Fdis	G	H	I	J	K	L	M	N	V	Z
860	534	385	1463	1149	314	600	635	537	26	1535	411	247	122	32	227	106	508	169	28
10.94	6.79	4.90	18.61	14.61	3.99	8.08	8.08	6.83	0.33	19.52	5.23	3.14	1.55	0.41	2.89	1.35	6.46	2.15	0.36

Abstracts and citances have the following truncated to max 2 levels unique IDs in common (first row -> number, second row -> %):
Total number of unique annotations = 1613

A	B	C	D	Dchem	Dent	E	F	Ffunc	Fdis	G	H	I	J	K	L	M	N	V	Z
154	176	47	301	126	175	40	112	94	1	555	55	56	7	0	10	2	74	24	0
9.55	10.91	2.91	18.66	7.81	10.85	6.94	6.94	5.83	0.06	34.41	3.41	3.47	0.43	0.00	0.62	0.12	4.59	1.49	0.00

Unique IDs (truncated to max 2 levels) found in abstracts but not in citances (first row -> number, second row -> %):
Total number of unique annotations = 311

A	B	C	D	Dchem	Dent	E	F	Ffunc	Fdis	G	H	I	J	K	L	M	N	V	Z
39	24	4	50	30	20	17	38	30	3	75	13	10	3	0	6	0	27	5	0
12.54	7.72	1.29	16.08	9.65	6.43	12.22	12.22	9.65	0.96	24.12	4.18	3.22	0.96	0.00	1.93	0.00	8.68	1.61	0.00

Unique IDs (truncated to max 2 levels) found in citances but not in abstracts (first row -> number, second row -> %):
Total number of unique annotations = 6249

A	B	C	D	Dchem	Dent	E	F	Ffunc	Fdis	G	H	I	J	K	L	M	N	V	Z
706	358	338	1162	1023	139	560	523	443	25	980	356	191	115	32	217	104	434	145	28
11.30	5.73	5.41	18.59	16.37	2.22	8.37	8.37	7.09	0.40	15.68	5.70	3.06	1.84	0.51	3.47	1.66	6.95	2.32	0.45

3. Counts Summary for 0-adjoining-citances and abstracts

Looking at all abstracts, the following unique IDs where found (first row -> number, second row -> %):
Total number of unique annotations = 2829

A	B	C	D	Dchem	Dent	E	F	Ffunc	Fdis	G	H	I	J	K	L	M	N	V	Z
228	259	51	673	195	478	65	153	127	4	1088	74	72	10	0	18	2	107	29	0
8.06	9.16	1.80	23.79	6.89	16.90	5.41	5.41	4.49	0.14	38.46	2.62	2.55	0.35	0.00	0.64	0.07	3.78	1.03	0.00

Looking at all citances with 0 adjoining citations, the following unique IDs where found (first row -> number, second row -> %):
Total number of unique annotations = 6014

A	B	C	D	Dchem	Dent	E	F	Ffunc	Fdis	G	H	I	J	K	L	M	N	V	Z
556	315	143	1529	655	874	369	305	264	8	1817	310	169	40	4	118	25	252	48	14
9.25	5.24	2.38	25.42	10.89	14.53	5.07	5.07	4.39	0.13	30.21	5.15	2.81	0.67	0.07	1.96	0.42	4.19	0.80	0.23

Abstracts and citances with 0 adjoining citations have the following unique IDs in common (first row -> number, second row -> %):
Total number of unique annotations = 1491

A	B	C	D	Dchem	Dent	E	F	Ffunc	Fdis	G	H	I	J	K	L	M	N	V	Z
136	129	32	332	99	233	26	62	47	0	615	42	36	4	0	5	0	53	19	0
9.12	8.65	2.15	22.27	6.64	15.63	4.16	4.16	3.15	0.00	41.25	2.82	2.41	0.27	0.00	0.34	0.00	3.55	1.27	0.00

Unique IDs found in abstracts but not in citances with 0 adjoining citations (first row -> number, second row -> %):
Total number of unique annotations = 1338

A	B	C	D	Dchem	Dent	E	F	Ffunc	Fdis	G	H	I	J	K	L	M	N	V	Z
92	130	19	341	96	245	39	91	80	4	473	32	36	6	0	13	2	54	10	0
6.88	9.72	1.42	25.49	7.17	18.31	6.80	6.80	5.98	0.30	35.35	2.39	2.69	0.45	0.00	0.97	0.15	4.04	0.75	0.00

Unique IDs found in citances with 0 adjoining citations but not in abstracts (first row -> number, second row -> %):
Total number of unique annotations = 4523

A	B	C	D	Dchem	Dent	E	F	Ffunc	Fdis	G	H	I	J	K	L	M	N	V	Z
420	186	111	1197	556	641	343	243	217	8	1202	268	133	36	4	113	25	199	29	14
9.29	4.11	2.45	26.46	12.29	14.17	5.37	5.37	4.80	0.18	26.58	5.93	2.94	0.80	0.09	2.50	0.55	4.40	0.64	0.31

Looking at all abstracts, the following truncated to max 2 levels unique IDs where found (first row -> number, second row -> %):
Total number of unique annotations = 1924

A	B	C	D	Dchem	Dent	E	F	Ffunc	Fdis	G	H	I	J	K	L	M	N	V	Z
193	200	51	351	156	195	57	150	124	4	630	68	66	10	0	16	2	101	29	0
10.03	10.40	2.65	18.24	8.11	10.14	7.80	7.80	6.44	0.21	32.74	3.53	3.43	0.52	0.00	0.83	0.10	5.25	1.51	0.00

Looking at all citances with 0 adjoining citations, the following truncated to max 2 levels unique IDs where found (first row -> number, second row -> %):
Total number of unique annotations = 3523

A	B	C	D	Dchem	Dent	E	F	Ffunc	Fdis	G	H	I	J	K	L	M	N	V	Z
378	217	132	716	496	220	252	248	208	8	855	200	115	36	3	81	25	206	48	11
10.73	6.16	3.75	20.32	14.08	6.24	7.04	7.04	5.90	0.23	24.27	5.68	3.26	1.02	0.09	2.30	0.71	5.85	1.36	0.31

Abstracts and citances with 0 adjoining citations have the following truncated to max 2 levels unique IDs in common (first row -> number, second row -> %):
Total number of unique annotations = 1234

A	B	C	D	Dchem	Dent	E	F	Ffunc	Fdis	G	H	I	J	K	L	M	N	V	Z
131	127	33	239	90	149	31	71	56	0	436	43	39	4	0	5	0	56	19	0
10.62	10.29	2.67	19.37	7.29	12.07	5.75	5.75	4.54	0.00	35.33	3.48	3.16	0.32	0.00	0.41	0.00	4.54	1.54	0.00

Unique IDs (truncated to max 2 levels) found in abstracts but not in citances with 0 adjoining citations (first row -> number, second row -> %):
Total number of unique annotations = 690

A	B	C	D	Dchem	Dent	E	F	Ffunc	Fdis	G	H	I	J	K	L	M	N	V	Z
62	73	18	112	66	46	26	79	68	4	194	25	27	6	0	11	2	45	10	0
8.99	10.58	2.61	16.23	9.57	6.67	11.45	11.45	9.86	0.58	28.12	3.62	3.91	0.87	0.00	1.59	0.29	6.52	1.45	0.00

Unique IDs (truncated to max 2 levels) found in citances with 0 adjoining citations but not in abstracts (first row -> number, second row -> %):
Total number of unique annotations = 2289

A	B	C	D	Dchem	Dent	E	F	Ffunc	Fdis	G	H	I	J	K	L	M	N	V	Z
247	90	99	477	406	71	221	177	152	8	419	157	76	32	3	76	25	150	29	11
10.79	3.93	4.33	20.84	17.74	3.10	7.73	7.73	6.64	0.35	18.30	6.86	3.32	1.40	0.13	3.32	1.09	6.55	1.27	0.48

4. Counts Summary for year/time effect considering MeSH treeIDs

Looking at all abstracts, the following unique IDs where found (first row -> number, second row -> %):
Total number of unique annotations = 2829

A	B	C	D	Dchem	Dent	E	F	Ffunc	Fdis	G	H	I	J	K	L	M	N	V	Z
228	259	51	673	195	478	65	153	127	4	1088	74	72	10	0	18	2	107	29	0
8.06	9.16	1.80	23.79	6.89	16.90	5.41	5.41	4.49	0.14	38.46	2.62	2.55	0.35	0.00	0.64	0.07	3.78	1.03	0.00

Looking at all citances appeared in year0 (same year as abstract), the following unique IDs where found (first row -> number, second row -> %):
Total number of unique annotations = 1131

A	B	C	D	Dchem	Dent	E	F	Ffunc	Fdis	G	H	I	J	K	L	M	N	V	Z
125	90	29	288	113	175	49	85	69	0	289	61	31	4	3	21	4	42	9	1
11.05	7.96	2.56	25.46	9.99	15.47	7.52	7.52	6.10	0.00	25.55	5.39	2.74	0.35	0.27	1.86	0.35	3.71	0.80	0.09

Looking at all citances appeared in year1 (1 year after the abstract), the following unique NEW IDs where found (first row -> number, second row -> %):
Total number of unique annotations = 3624

A	B	C	D	Dchem	Dent	E	F	Ffunc	Fdis	G	H	I	J	K	L	M	N	V	Z
286	283	98	760	337	423	193	202	174	7	1217	152	95	19	9	86	21	146	52	5
7.89	7.81	2.70	20.97	9.30	11.67	5.57	5.57	4.80	0.19	33.58	4.19	2.62	0.52	0.25	2.37	0.58	4.03	1.43	0.14

Looking at all citances appeared in year2 (2 years after the abstract), the following unique NEW IDs where found (first row -> number, second row -> %):
Total number of unique annotations = 2866

A	B	C	D	Dchem	Dent	E	F	Ffunc	Fdis	G	H	I	J	K	L	M	N	V	Z
181	215	95	657	343	314	220	212	193	7	701	144	110	32	10	83	22	152	30	2
6.32	7.50	3.31	22.92	11.97	10.96	7.40	7.40	6.73	0.24	24.46	5.02	3.84	1.12	0.35	2.90	0.77	5.30	1.05	0.07

Looking at all citances appeared in year3 (3 years after the abstract), the following unique NEW IDs where found (first row -> number, second row -> %):
Total number of unique annotations = 1946

A	B	C	D	Dchem	Dent	E	F	Ffunc	Fdis	G	H	I	J	K	L	M	N	V	Z
84	143	90	455	291	164	176	135	119	6	441	119	50	32	5	63	23	92	29	9
4.32	7.35	4.62	23.38	14.95	8.43	6.94	6.94	6.12	0.31	22.66	6.12	2.57	1.64	0.26	3.24	1.18	4.73	1.49	0.46

Looking at all citances appeared in year4+ (4+ years after the abstract), the following unique NEW IDs where found (first row -> number, second row -> %):
Total number of unique annotations = 6615

A	B	C	D	Dchem	Dent	E	F	Ffunc	Fdis	G	H	I	J	K	L	M	N	V	Z
355	504	195	1450	815	635	426	449	411	8	1874	357	197	78	8	156	62	410	68	26
5.37	7.62	2.95	21.92	12.32	9.60	6.79	6.79	6.21	0.12	28.33	5.40	2.98	1.18	0.12	2.36	0.94	6.20	1.03	0.39

Looking at all unique IDs that the abstracts and citances appeared in year0 (same year as abstract) have in common (first row -> number, second row -> %):
Total number of unique annotations = 419

A	B	C	D	Dchem	Dent	E	F	Ffunc	Fdis	G	H	I	J	K	L	M	N	V	Z
37	41	7	98	31	67	4	20	16	0	168	16	9	1	0	0	1	12	5	0
8.83	9.79	1.67	23.39	7.40	15.99	4.77	4.77	3.82	0.00	40.10	3.82	2.15	0.24	0.00	0.00	0.24	2.86	1.19	0.00

Looking at all NEW unique IDs that the abstracts and citances appeared in year1 (1 year after the abstract) have in common (first row -> number, second row -> %):
Total number of unique annotations = 807

A	B	C	D	Dchem	Dent	E	F	Ffunc	Fdis	G	H	I	J	K	L	M	N	V	Z
68	97	27	139	40	99	11	38	32	1	335	18	26	1	0	3	1	31	12	0
8.43	12.02	3.35	17.22	4.96	12.27	4.71	4.71	3.97	0.12	41.51	2.23	3.22	0.12	0.00	0.37	0.12	3.84	1.49	0.00

Looking at all NEW unique IDs that the abstracts and citances appeared in year2 (2 years after the abstract) have in common (first row -> number, second row -> %):
Total number of unique annotations = 354

A	B	C	D	Dchem	Dent	E	F	Ffunc	Fdis	G	H	I	J	K	L	M	N	V	Z
18	26	12	86	28	58	12	23	18	0	137	10	10	1	0	2	0	14	3	0
5.08	7.34	3.39	24.29	7.91	16.38	6.50	6.50	5.08	0.00	38.70	2.82	2.82	0.28	0.00	0.56	0.00	3.95	0.85	0.00

Looking at all NEW unique IDs that the abstracts and citances appeared in year3 (3 years after the abstract) have in common (first row -> number, second row -> %):
Total number of unique annotations = 135

A	B	C	D	Dchem	Dent	E	F	Ffunc	Fdis	G	H	I	J	K	L	M	N	V	Z
5	11	0	40	13	27	1	11	9	0	50	3	3	2	0	4	0	3	2	0
3.70	8.15	0.00	29.63	9.63	20.00	8.15	8.15	6.67	0.00	37.04	2.22	2.22	1.48	0.00	2.96	0.00	2.22	1.48	0.00

Looking at all NEW unique IDs that the abstracts and citances appeared in year4+ (4+ years after the abstract) have in common (first row -> number, second row -> %):
Total number of unique annotations = 225

A	B	C	D	Dchem	Dent	E	F	Ffunc	Fdis	G	H	I	J	K	L	M	N	V	Z
13	29	1	38	23	15	10	9	8	0	95	6	4	1	0	3	0	14	2	0
5.78	12.89	0.44	16.89	10.22	6.67	4.00	4.00	3.56	0.00	42.22	2.67	1.78	0.44	0.00	1.33	0.00	6.22	0.89	0.00

5. Counts Summary considering year/time effect for truncated MeSH treeIDs

Looking at all abstracts, the following unique IDs where found (first row -> number, second row -> %):
Total number of unique annotations = 1924

A	B	C	D	Dchem	Dent	E	F	Ffunc	Fdis	G	H	I	J	K	L	M	N	V	Z
193	200	51	351	156	195	57	150	124	4	630	68	66	10	0	16	2	101	29	0
10.03	10.40	2.65	18.24	8.11	10.14	7.80	7.80	6.44	0.21	32.74	3.53	3.43	0.52	0.00	0.83	0.10	5.25	1.51	0.00

Looking at all citances appeared in year0 (same year as abstract), the following unique IDs where found (first row -> number, second row -> %):
Total number of unique annotations = 778

A	B	C	D	Dchem	Dent	E	F	Ffunc	Fdis	G	H	I	J	K	L	M	N	V	Z
84	62	29	156	90	66	35	73	60	0	193	46	27	4	3	13	4	39	9	1
10.80	7.97	3.73	20.05	11.57	8.48	9.38	9.38	7.71	0.00	24.81	5.91	3.47	0.51	0.39	1.67	0.51	5.01	1.16	0.13

Looking at all citances appeared in year1 (1 year after the abstract), the following unique NEW IDs where found (first row -> number, second row -> %):
Total number of unique annotations = 2046

A	B	C	D	Dchem	Dent	E	F	Ffunc	Fdis	G	H	I	J	K	L	M	N	V	Z
210	146	87	345	236	109	147	166	139	7	497	101	68	18	7	56	21	123	52	2
10.26	7.14	4.25	16.86	11.53	5.33	8.11	8.11	6.79	0.34	24.29	4.94	3.32	0.88	0.34	2.74	1.03	6.01	2.54	0.10

Looking at all citances appeared in year2 (2 years after the abstract), the following unique NEW IDs where found (first row -> number, second row -> %):
Total number of unique annotations = 1563

A	B	C	D	Dchem	Dent	E	F	Ffunc	Fdis	G	H	I	J	K	L	M	N	V	Z
127	105	75	300	241	59	135	143	124	7	273	82	64	29	9	49	22	118	30	2
8.13	6.72	4.80	19.19	15.42	3.77	9.15	9.15	7.93	0.45	17.47	5.25	4.09	1.86	0.58	3.13	1.41	7.55	1.92	0.13

Looking at all citances appeared in year3 (3 years after the abstract), the following unique NEW IDs where found (first row -> number, second row -> %):
Total number of unique annotations = 1089

A	B	C	D	Dchem	Dent	E	F	Ffunc	Fdis	G	H	I	J	K	L	M	N	V	Z
69	69	61	230	199	31	107	90	74	6	191	61	25	24	5	36	23	62	29	7
6.34	6.34	5.60	21.12	18.27	2.85	8.26	8.26	6.80	0.55	17.54	5.60	2.30	2.20	0.46	3.31	2.11	5.69	2.66	0.64

Looking at all citances appeared in year4+ (4+ years after the abstract), the following unique NEW IDs where found (first row -> number, second row -> %):
Total number of unique annotations = 2168

A	B	C	D	Dchem	Dent	E	F	Ffunc	Fdis	G	H	I	J	K	L	M	N	V	Z
181	141	128	430	381	49	166	163	140	6	380	121	63	47	8	73	36	166	49	16
8.35	6.50	5.90	19.83	17.57	2.26	7.52	7.52	6.46	0.28	17.53	5.58	2.91	2.17	0.37	3.37	1.66	7.66	2.26	0.74

Looking at all unique IDs that the abstracts and citances appeared in year0 (same year as abstract) have in common (first row -> number, second row -> %):
Total number of unique annotations = 373

A	B	C	D	Dchem	Dent	E	F	Ffunc	Fdis	G	H	I	J	K	L	M	N	V	Z
35	37	7	77	25	52	7	24	19	0	140	17	10	1	0	0	1	12	5	0
9.38	9.92	1.88	20.64	6.70	13.94	6.43	6.43	5.09	0.00	37.53	4.56	2.68	0.27	0.00	0.00	0.27	3.22	1.34	0.00

Looking at all NEW unique IDs that the abstracts and citances appeared in year1 (1 year after the abstract) have in common (first row -> number, second row -> %):
Total number of unique annotations = 683

A	B	C	D	Dchem	Dent	E	F	Ffunc	Fdis	G	H	I	J	K	L	M	N	V	Z
69	80	28	115	47	68	15	44	39	1	238	17	28	1	0	4	1	31	12	0
10.10	11.71	4.10	16.84	6.88	9.96	6.44	6.44	5.71	0.15	34.85	2.49	4.10	0.15	0.00	0.59	0.15	4.54	1.76	0.00

Looking at all NEW unique IDs that the abstracts and citances appeared in year2 (2 years after the abstract) have in common (first row -> number, second row -> %):
Total number of unique annotations = 268

A	B	C	D	Dchem	Dent	E	F	Ffunc	Fdis	G	H	I	J	K	L	M	N	V	Z
17	27	11	55	24	31	11	25	20	0	80	11	10	2	0	1	0	15	3	0
6.34	10.07	4.10	20.52	8.96	11.57	9.33	9.33	7.46	0.00	29.85	4.10	3.73	0.75	0.00	0.37	0.00	5.60	1.12	0.00

Looking at all NEW unique IDs that the abstracts and citances appeared in year3 (3 years after the abstract) have in common (first row -> number, second row -> %):
Total number of unique annotations = 104

A	B	C	D	Dchem	Dent	E	F	Ffunc	Fdis	G	H	I	J	K	L	M	N	V	Z
7	10	0	27	13	14	1	7	5	0	33	3	4	2	0	2	0	6	2	0
6.73	9.62	0.00	25.96	12.50	13.46	6.73	6.73	4.81	0.00	31.73	2.88	3.85	1.92	0.00	1.92	0.00	5.77	1.92	0.00

Looking at all NEW unique IDs that the abstracts and citances appeared in year4+ (4+ years after the abstract) have in common (first row -> number, second row -> %):
Total number of unique annotations = 170

A	B	C	D	Dchem	Dent	E	F	Ffunc	Fdis	G	H	I	J	K	L	M	N	V	Z
11	22	1	27	17	10	6	12	11	0	64	7	4	1	0	3	0	10	2	0
6.47	12.94	0.59	15.88	10.00	5.88	7.06	7.06	6.47	0.00	37.65	4.12	2.35	0.59	0.00	1.76	0.00	5.88	1.18	0.00

6. Overall year/time effect for 0adjoining citances

PER TREE ID

YEAR	ANNOTATIONS	UNIQUE_ANNOTATIONS_NOT_APPEARED_BEFORE	UNIQUE_ANNOTATIONS_IN_COMMON_WITH_ABSTRACTS
0	793	793	381
1	2666	2083	692
2	3366	1583	275
3	3234	1096	107
4+	5312	2178	172
		total =	1627 of 1962 found in abstracts

PER TRUNCATED TREE ID

YEAR	ANNOTATIONS	UNIQUE_ANNOTATIONS_NOT_APPEARED_BEFORE	UNIQUE_ANNOTATIONS_IN_COMMON_WITH_ABSTRACTS
0	1145	1145	427
1	4610	3360	822
2	6096	2885	361
3	5737	1952	138
4+	19823	6641	227
		total =	1975 of 2867 found in abstracts

CITANCES WITH 0 ADJOINING CITATIONS VS ABSTRACTS

FILE	ANNOTATIONS
abstracts treeIDS	2867
adj0 treeIDs	6075
abstracts_adj0 common treeIDs	1516
abstracts trunc_treeIDs	1962
adj0 trunc_treeIDs	3584
abstracts_adj0 common trunc_treeIDs	1259
